# Beyond Tumor Immunity: The Disruption of Endocrine and Infectious Homeostasis by Immune Checkpoint Inhibitors

**DOI:** 10.3390/ijms262311619

**Published:** 2025-11-30

**Authors:** Ema Schönberger, Luka Švitek, Barbara Grubišić, Tara Cvijić Perić, Romana Marušić, Nika Vlahović Vlašić, Tomislav Kizivat, Silvija Canecki Varžić, Lorna Stemberger Marić, Ines Bilić Ćurčić

**Affiliations:** 1 Department of Endocrinology, Internal Medicine Clinic, University Hospital Centre Osijek, 31000 Osijek, Croatia; 2 Faculty of Medicine Osijek, J. J. Strossmayer University of Osijek, 31000 Osijek, Croatia; 3 Department of Medical Microbiology and Infectious Diseases, Faculty of Medicine Osijek, J. J. Strossmayer University of Osijek, 31000 Osijek, Croatia; 4 Clinic of Infectious Diseases, University Hospital Centre Osijek, 31000 Osijek, Croatia; 5 Clinic of Oncology, University Hospital Centre Osijek, 31000 Osijek, Croatia; 6 National Memorial Hospital “Dr. Juraj Njavro” Vukovar, 32000 Vukovar, Croatia; 7 Clinical Institute of Nuclear Medicine and Radiation Protection, University Hospital Osijek, 31000 Osijek, Croatia; 8 Department for Nuclear Medicine, Faculty of Medicine, J. J. Strossmayer University of Osijek, 31000 Osijek, Croatia; 9 Department of Pathophysiology, Faculty of Medicine Osijek, J. J. Strossmayer University of Osijek, 31000 Osijek, Croatia; 10 Department of Pediatric Infectious Diseases, University Hospital for Infectious Diseases, 10000 Zagreb, Croatia; 11 Department of Infectious Diseases, School of Medicine, University of Zagreb, 10000 Zagreb, Croatia; 12 Department of Pharmacology, Faculty of Medicine Osijek, J. J. Strossmayer University of Osijek, 31000 Osijek, Croatia

**Keywords:** immune checkpoint inhibitors, immune-related adverse effects, infections, endocrine dysfunction

## Abstract

Immune checkpoint inhibitors (ICIs) have revolutionized cancer treatment by reactivating T cell-mediated anti-tumor immunity. However, this enhanced immune activity can lead to immune-related adverse events (irAEs). This narrative review focuses on endocrine irAEs, including thyroid dysfunction, hypophysitis, adrenal insufficiency, and diabetes mellitus. It also explores infectious complications and their underlying mechanisms. These mechanisms include immune dysregulation resulting directly from ICI-induced T-cell activation and indirectly from the immunosuppressive therapies used to treat irAEs. Furthermore, potential role of endocrine irAEs in predisposing patients to infectious complications is analyzed. The objective is to provide non-oncology specialists with the clinical insight necessary to recognize and manage these complex side effects. This narrative review synthesizes current literature on the diagnosis, management, and pathophysiology of endocrine irAEs and infections associated with different classes of ICIs (anti-CTLA-4, anti-PD-1, and anti-PD-L1). Endocrine irAEs are common, with incidence varying by ICI type; combination therapies pose the highest risk. Thyroid dysfunction is the most frequent, followed by hypophysitis, which often leads to permanent secondary adrenal insufficiency. ICI-induced diabetes mellitus is a rare but serious complication, frequently presenting as diabetic ketoacidosis. ICIs are believed to induce a distinct array of infections resulting from immunological dysregulation, unrelated to immunosuppressive medication. The phenomenon is increasingly called ICI therapy-induced dysregulated immunity. Moreover, evidence suggests that endocrine irAEs can compromise immune function and lead to a significantly higher risk of bacterial and fungal infections. Identifying infections that imitate irAEs is particularly important because the therapy is significantly distinct. Greater interdisciplinary awareness is crucial for the early recognition and appropriate management of both the endocrine and infectious complications, ultimately improving the safety and outcomes for patients receiving immunotherapy.

## 1. Introduction

Recent advances in cancer research have introduced new concepts in oncology treatment. One such concept is the use of immune checkpoint inhibitors (ICIs), whose primary goal is to reactivate T cell–mediated immune responses against tumor cells. Currently, three groups of ICIs are in clinical use: programmed cell death protein 1 (PD-1) inhibitors, programmed death-ligand 1 (PD-L1) inhibitors, and cytotoxic T-lymphocyte-associated protein 4 (CTLA-4) inhibitors. PD-1 and CTLA-4 are co-inhibitory receptors on the surface of T cells, primarily responsible for negatively regulating T cell–mediated immune responses. Various cancer cell products exploit these receptors to suppress the immune response and evade elimination by the host immune system. ICIs target these receptors, thereby preventing cancer cells from using them to their advantage [1,2,3].

Although this form of therapy is often associated with a more favorable toxicity profile compared to traditional cancer treatments, several adverse effects must not be overlooked. In this narrative review, we will explore immune-related adverse events (irAEs) associated with ICIs, with a particular focus on endocrine irAEs, as well as common infectious complications related to ICI therapy.

Clinicians prescribing immune checkpoint inhibitors are often not the same as those managing their adverse effects. For instance, a patient receiving ICIs who develops endocrine-related symptoms may initially present to the emergency department. If fever is among the presenting symptoms, the patient may instead be referred to an infectious diseases clinic, particularly when an infectious complication related to ICI therapy is suspected or cannot be excluded. In such cases, the immune-related etiology may not be immediately recognized, underscoring the importance of interdisciplinary awareness. Therefore, this narrative review aims to provide a comprehensive overview of current knowledge on ICI-associated adverse events, with the goal of equipping non-oncology specialists with the necessary clinical insight to recognize and appropriately evaluate these complications.

## 2. Overview of Immune Checkpoint Inhibitors and Immune-Related Adverse Events

The discovery of immunotherapy has revolutionized the treatment of malignant diseases, significantly improved the quality of life, and prolonged the survival of cancer patients [4]. As already mentioned, there are three groups of ICIs.

CTLA4 is a receptor protein on T-lymphocytes which inhibits the interaction between CD80/CD86 molecules on antigen-presenting cells and CD28 molecules on T-lymphocytes [4]. This activity suppresses the cytotoxic activity of T-lymphocytes and stimulates regulatory T-lymphocytes (Treg) which is why the inhibition of this activity has antitumor effect [4]. In clinical practice, the most commonly used is ipilimumab which was initially approved for the treatment of melanoma [5]. Today it is also used in the treatment of other tumors (most often with nivolumab). Tremelimumab is also increasingly used, most often with durvalumab in the treatment of lung cancer and hepatocellular carcinoma [6,7].

PD-1 protein is found on activated T-lymphocytes and one of its ligands, PD-L1 is found on tumor cells, immune cells, endothelial cells and numerous others [8]. PD-1 and PD-L1 inhibitors that block this interaction enable T-lymphocytes to have a cytotoxic antitumor effect [4]. The most important PD-L1 inhibitors are atezolizumab, durvalumab and avelumab, while the PD-1 inhibitors are pembrolizumab, nivolumab and cemiplimab, all of which are used in the treatment of numerous solid tumors. All checkpoint inhibitors initially proved successful in the treatment of metastatic cancer, but according to recent clinical trials, many of them also show extremely successful activity in both adjuvant and neoadjuvant treatment [4]. Although checkpoint inhibitors show good efficacy, not all patients respond, and resistance may develop through loss of tumor antigens, MHC molecules, or impaired IFN-γ signaling [9]. Novel agents are being developed to target innate immunity [10]. While generally safe, checkpoint inhibitors can cause irAEs involving multiple organs, usually reversible but sometimes permanent or life-threatening, resembling autoimmune disorders due to immune dysregulation.

Some of them are the production of autoantibodies (the same or similar as in autoimmune diseases), increased levels of different T-lymphocyte clones in the blood and tissues, and increased production of proinflammatory cytokines such as interleukins [11]. Interleukin-6 stood out particularly, showing significant elevation during adverse events, while lower baseline levels prior to therapy were associated with a higher likelihood of developing irAEs [11,12]. When it comes to T-lymphocytes, decreased levels of Treg and increased levels of CD4+ and CD8+ T-lymphocytes, and especially CD4 effector memory T cells contribute to the occurrence of adverse events [13]. It is also believed that the specificities of each organism also play a role in the occurrence of these irAEs, such as an imbalance in the intestinal microbiota [11].

According to some studies, irAEs of all grades occur in 90% of patients treated with CTLA4 inhibitors and in 70% of patients treated with PD-1/PD-L1 inhibitors and are more common when these two groups are combined (especially high—grade irAEs) [14,15]. Data from clinical trials vary somewhat, but according to meta-analyses, the most common irAEs are skin disorders (most commonly rash and eczema), then gastrointestinal (nausea, decreased appetite, diarrhea, colitis), followed by endocrinological [11]. Less common but potentially severe adverse events such as myositis, rhabdomyolysis, nephritis, pneumonitis, and myocarditis can also occur [16]. Ophthalmological adverse events and neurotoxicity are also described [11].

Diagnosis of irAEs depends on the organ system involved and may require tissue biopsies (in the case of skin adverse events and colitis), imaging methods, monitoring of laboratory parameters, and monitoring of certain hormone levels. Treatment options for irAEs are currently very limited and include only temporary or permanent discontinuation of therapy for grade 3–4 adverse events, symptomatic treatment, immunosuppression with corticosteroids or other immunosuppressants (such as the TNF-α blocker infliximab), and possibly hormone replacement therapy [17]. The dosage of corticosteroids and other immunosuppressants depends on the severity of the adverse event and the organ system involved. The guidelines of the leading oncology societies in the world, such as the National Comprehensive Cancer Network, American Society of Clinical Oncology, and European Society for Medical Oncology are most often used in the treatment of adverse events [14,18,19]. Development of irAEs has been associated with significantly longer overall and progression-free survival across most solid tumors [20]. Several blood-based markers, including the systemic immune-inflammation index, neutrophil-to-lymphocyte and platelet-to-lymphocyte ratios, low LDH and IL-6, and elevated CRP, have been linked to both irAEs and improved prognosis [21,22,23]. While their role in clinical practice remains under investigation, such biomarkers may help identify patients at risk for adverse events and optimize immunotherapy outcomes.

Endocrine irAEs represent a unique clinical challenge, as they disrupt the critical homeostatic interplay between the endocrine and immune systems. A central thesis of this review is the exploration of these mechanistic links. We will posit and subsequently elaborate on the mechanistic hypotheses explaining how specific endocrine dysfunctions modulate host immune responses. Specifically, we will examine how cortisol deficiency—a direct consequence of adrenal insufficiency—may impair adequate T-cell function and the systemic stress response. Similarly, we will address how thyroid hormone dysregulation (both hyper- and hypothyroidism) can alter neutrophil and macrophage function, thereby impacting the efficacy of the inflammatory response. Concurrently, we will analyze how the hyperglycemic state induced by ICI-related diabetes mellitus demonstrably alters both innate and adaptive host defenses, thereby creating a distinct vulnerability to infection.

## 3. Endocrine Immune-Related Adverse Events

### 3.1. Thyroid Dysfunction

The immune-related adverse events affecting the thyroid are among the most common endocrine manifestations associated with immune checkpoint inhibitors, most frequently presenting as hypothyroidism, hyperthyroidism, or transient thyroiditis [24]. Unlike hypophysitis, which predominantly affects males, thyroid dysfunction related to immune checkpoint inhibitors appears to be more common in females [25]. The highest incidence rates of thyroid dysfunction have been observed with combination immune checkpoint inhibitor therapy, with estimates ranging between 8.0% and 16.4%. In comparison, monotherapy with anti-PD-1 agents results in lower frequencies (2.8–8.5%), followed by anti-PD-L1 (0.6–6.0%) and anti-CTLA-4 inhibitors (0.2–5.2%) [24,26,27].

Thyrotoxicosis related to thyroid irAEs typically emerges between 2 and 6 weeks following ICI initiation and often precedes the onset of hypothyroidism, which generally develops around week 12 [28]. The onset of hyperthyroidism typically occurs earlier with combination immune checkpoint blockade, with a median time of approximately 21 days, compared to around 47 days in patients receiving anti-PD-1 monotherapy [29]. Moreover, although both nivolumab and pembrolizumab are classified as anti-PD-1 antibodies, they appear to exhibit different thyroid toxicity profiles: nivolumab is more commonly associated with hypothyroidism, whereas pembrolizumab tends to be linked with a higher incidence of hyperthyroidism [30]. Patients with thyrotoxicosis may experience tachycardia, unintentional weight loss, fatigue, and diarrhea, while hypothyroidism is typically characterized by bradycardia, weight gain, fatigue, and constipation [31,32].

**Figure 1:**
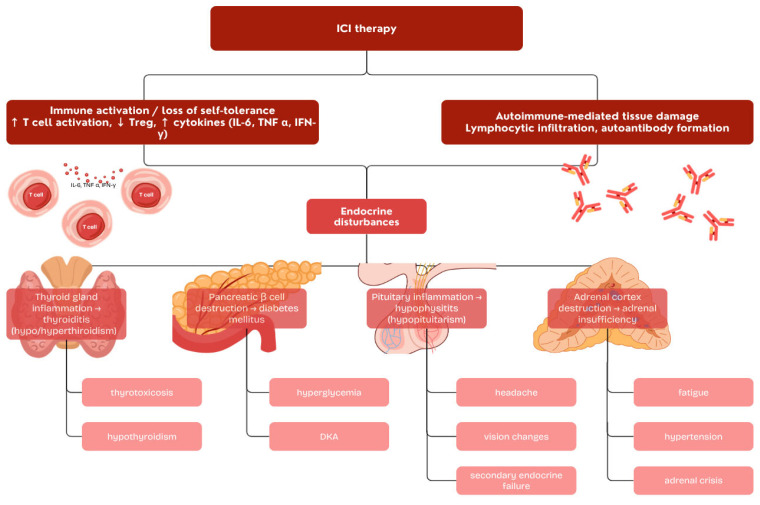
Overview of immune checkpoint inhibitors and endocrine immune-related adverse events.

The predominant underlying mechanism appears to be destructive thyroiditis, driven by immune-mediated acute inflammation and subsequent thyroid follicular cell damage. ICI therapy facilitates the development of autoimmune toxicity mainly through T cell activation, and histopathological analyses have demonstrated an intra-thyroidal predominance of CD8^+^ cytotoxic T lymphocytes and double-negative CD4^−^CD8^−^ T cells (Figure 1) [32,33].

Baseline assessment of thyroid-stimulating hormone (TSH) and free T4 (fT4) is recommended prior to initiating ICI therapy to exclude preexisting thyroid dysfunction. During treatment, thyroid function (typically TSH and fT4) should be monitored every 4–6 weeks, or more frequently if clinically indicated [25]. In cases of thyrotoxicosis, current guidelines recommend testing for TSH receptor antibodies to evaluate for autoimmune hyperthyroidism. Conversely, in hypothyroidism, assessment of thyroid peroxidase antibodies is advised, as their presence supports an autoimmune origin [28,32].

In most cases, thyrotoxicosis warrants only clinical observation. For patients with moderate to severe symptoms, β-blockers may be used to alleviate adrenergic manifestations. In contrast, hypothyroidism that follows thyrotoxicosis should be managed with levothyroxine replacement, as it often progresses to overt and persistent hypothyroidism. Graves’ disease requires treatment with anti-thyroid drugs [25,28]. In most cases, thyroid dysfunction does not necessitate discontinuation of immune checkpoint inhibitor therapy. With appropriate thyroid hormone replacement, patients can safely continue immunotherapy [28].

### 3.2. Hypophysitis

After thyroid dysfunction, hypophysitis is the second most common immune-mediated endocrinopathy. Due to its lack of specificity in clinical manifestations, misdiagnosis and delayed diagnosis can lead to poor prognosis for patients.

The risk of developing hypophysitis is strongly correlated with the specific class of ICI used, with the highest incidence with combined anti-CTLA-4 and anti-PD-1/PD-L1 agents, with rates from 6.4% to 19% [34,35]. The incidence of immune-related hypophysitis associated with CTLA-4 inhibitor and PD-1/PD-L1 inhibitor monotherapy is much lower than that of combination therapy, accounting for 5% and 1%, respectively [30,36]. For anti-CTLA-4 inhibitors, higher doses are associated with a higher incidence of hypophysitis [37]. Demographically, there is a notable male predominance and patients who develop hypophysitis tend to be older than those who do not [38].

ICI-induced hypophysitis is an autoimmune disorder driven by T-cells attacking pituitary cells. The mechanisms differ markedly between ICI classes. CTLA-4 is expressed on normal pituitary cells, allowing anti-CTLA-4 antibodies to bind directly, triggering complement activation and antibody-dependent cytotoxicity [39]. This localized inflammation explains the high incidence, fast onset, and frequent pituitary enlargement seen with these agents. In contrast, the mechanism for anti-PD-1/PD-L1 blockade is more indirect and it is unknown if PD-1 or PD-L1 is expressed on pituitary cells [40,41]. Furthermore, anti-PD-1 antibodies (typically IgG4) are poor activators of complement or cytotoxicity. The pathophysiology is thought to be related to peripherally activated T cells that are found in the pituitary gland (Figure 1). This less intense process is probably the reason for the lower incidence, later onset and characteristic lack of pituitary enlargement with these drugs.

The timing of onset of hypophysitis is earliest with combination therapy with median 1–3 months, followed with anti-CTLA-4 monotherapy (median 9–11 weeks), and usually later with anti-PD-1/PD-L1 monotherapy with median 4–7 months. The clinical presentation is often dominated by nonspecific symptoms like profound fatigue, weakness, and nausea [37]. The CTLA-4 phenotype is often marked by severe headaches due to pituitary mass effect. The pattern of hormone loss also differs. Secondary adrenal insufficiency is the most common and clinically urgent deficit of all types. In the PD-1/PD-L1 phenotype, it is often the only deficit, while central hypothyroidism and secondary hypogonadism are very common in the CTLA-4 phenotype. Posterior pituitary involvement (diabetes insipidus) is exceedingly rare [42]. Diagnosis is primarily biochemical, as radiological findings may be absent. Regular screening with baseline pituitary tests and monitoring during the first 6 months of ICI therapy is recommended [43,44,45]. Biochemically, target organ hormone deficiency with low or inappropriately normal pituitary hormone suggests hypophysitis. Pituitary MRI with contrast can reveal enlargement and stalk thickening, especially with anti-CTLA-4 agents, but may appear normal in PD-1/PD-L1–related cases; therefore, a normal MRI does not exclude the diagnosis [42].

According to the current European Society for Medical Oncology (ESMO) guidelines, the management of hypophysitis primarily relies on hormone replacement therapy (hydrocortisone or prednisolone). The use of high-dose corticosteroids (e.g., prednisone 1 mg/kg/day or equivalent) is reserved only for patients with severe manifestations, including compressive symptoms such as headache, visual disturbance, or adrenal crisis [19]. For stable patients, chronic oral replacement (e.g., hydrocortisone 15–20 mg daily in divided doses) is initiated. Patient education on “sick day rules” and use of an emergency hydrocortisone injection kit is mandatory [46]. In patients with concurrent adrenal insufficiency and hypothyroidism, glucocorticoid replacement must be started first to prevent precipitating an adrenal crisis. Central hypothyroidism is treated with levothyroxine, with the dose titrated to maintain free T4 in the upper half of the reference range [47]. However, the use of high-dose, immunosuppressive glucocorticoids is controversial. A retrospective study from 2015 failed to demonstrate any beneficial effect of high-dose steroids on either hypophysitis or malignancy outcomes in patients treated with ipilimumab [48]. The development of hypophysitis is not an absolute contraindication to continuing ICI therapy. The standard approach is to temporarily withhold ICI during the acute phase. Once the patient is stable on hormone replacement, ICI therapy can generally be safely resumed.

The hormonal deficits from ICI-induced hypophysitis are often permanent. Recovery of the corticotroph axis is very rare, and secondary adrenal insufficiency is considered a lifelong condition for the majority of patients. The prognosis for the thyroid and gonadal axes is slightly better, but recovery still occurs in less than half of affected patients.

### 3.3. Adrenal Dysfunction

Primary adrenal insufficiency (AI) associated with ICI therapy is a rare but clinically important irAE. It occurs in approximately 1–2% of patients receiving anti-PD-1 monotherapy, with incidence rising significantly—up to 4–9% in those treated with a combination of anti-PD-1 and anti-CTLA-4 agents [49,50]. The median time to onset is variable, typically around 10 weeks after treatment initiation, though reported ranges span from 2.5 to 4.3 months depending on the ICI regimen [51]. Data from the World Health Organization VigiBase identified 451 cases of primary AI among 50,000 irAEs reported since 2008, of which 46 were confirmed as definitive [52]. The pathophysiology of ICI-induced primary adrenal insufficiency is not fully defined but likely involves loss of immune tolerance and T-cell–mediated adrenal damage [53]. Possible mechanisms include molecular mimicry, pre-existing autoreactive T cells, 21-hydroxylase autoantibodies, and HLA genotypes associated with autoimmune endocrinopathies (Figure 1) [54,55].

Clinically, ICI-induced AI may present acutely with adrenal crisis or more insidiously with non-specific symptoms. It should be suspected in patients with unexplained hypotension, fever, abdominal pain, persistent nausea or vomiting, altered mental status, hyponatraemia, hypoglycaemia, or hyperkalaemia even in the absence of known adrenal or pituitary disease [49,56].

Diagnostic evaluation begins with morning serum cortisol and adrenocorticotropic hormone (ACTH) measurement. Low cortisol with elevated ACTH confirms primary AI, whereas low cortisol with low or inappropriately normal ACTH suggests secondary AI. When results are inconclusive, a 250 µg Synacthen (cosyntropin) stimulation test can assess adrenal reserve [25]. Imaging supports etiological differentiation: pituitary MRI may reveal hypophysitis in secondary AI, while adrenal CT or MRI may show glandular atrophy or enlargement, aiding distinction between autoimmune destruction and metastatic infiltration [25,57]. Routine screening for 21-hydroxylase antibodies is recommended, as their presence supports autoimmune adrenal cortex destruction [25].

Management of adrenal crisis requires prompt intravenous administration of 100 mg hydrocortisone, followed by appropriate fluid resuscitation and 200 mg of hydrocortisone/24 h (via continuous iv therapy or 6 hourly injection). Long-term management involves daily oral hydrocortisone (15–25 mg/day) in two or three divided doses. In cases of primary AI, fludrocortisone is added to replace mineralocorticoid activity. Adrenal insufficiency does not typically necessitate permanent discontinuation of ICIs. Once hormone deficits are adequately replaced, immunotherapy may be safely continued, especially in patients showing favorable oncologic response [58].

### 3.4. Immune Checkpoint Inhibitor-Induced Diabetes Mellitus

ICI-induced diabetes mellitus (ICI-DM) has been reported to occur in approximately 0.2–1.9% of patients treated with ICI and is being recognized and diagnosed with increasing frequency [35,59,60,61,62]. Exacerbation of underlying type 2 diabetes may also occur, though frequency of this is unknown. To diagnose ICI-DM, there should be clear evidence of marked hyperglycemia resulting from the reduction in insulin production following ICI therapy. Although the presence of type 1 diabetes mellitus (T1DM) autoantibodies supports the diagnosis, they are not always present [63]. The incidence rates of ICI-DM vary depending on the inhibitor used. Therapies targeting the PD-1/PD-L1 pathway, whether administered as monotherapy or in combination, are implicated in the vast majority, about 97%, of reported cases. Conversely, the development of ICI-DM in patients receiving anti-CTLA-4 monotherapy is very rare [61,64,65]. It should be emphasized that the development of ICI-induced DM is mainly observed in melanoma. This observation is likely due to the initial approval of PD-1 inhibitors for melanoma, which resulted in a larger patient population being treated for this disease than for other malignancies [66].

The pathogenesis of ICI-DM is based on the disruption of the PD-1/PD-L1 immune checkpoint, a crucial signaling pathway for the maintenance of peripheral self-tolerance [67]. Pancreatic β-cells can upregulate the expression of PD-L1, especially under inflammatory conditions, to protect themselves from autoreactive T-cells. By blocking this protective PD-1/PD-L1 interaction, ICIs release pre-existing or newly activated autoreactive T-cells, leading to targeted destruction of β-cells (Figure 1) [63,68]. ICI-DM is characterized by CD8+ T-cell–mediated insulitis, similar to classic T1DM [69]. Unlike T1DM, autoantibodies are less frequent (30–50% vs. ~90%) [70,71,72], while genetic predisposition, especially HLA-DR4, appears more prominent [70,73].

The time from ICI initiation to diagnosis of ICI-DM can vary widely. According to the reports, the median time to onset ranges from 10 to 29 weeks [66,70,72,74]. However, ICI-DM can be a delayed immune-related adverse event, with some cases emerging 6 months or more after the conclusion of therapy [70,75]. The most common and dramatic clinical presentation is diabetic ketoacidosis (DKA), which occurs in up to 76% of patients at the time of diagnosis [66,76]. This high incidence is a consequence of the rapid, almost complete destruction of β-cells combined with a possible delay in recognizing early symptoms. It is important to educate patients about the potential symptoms of ICI-DM, including polyuria, polydipsia, fatigue and polyphagia [47]. Due to the fulminant nature of the disease, the glycated hemoglobin (HbA1c) value at presentation is often disproportionately low for the degree of acute hyperglycemia. The characteristic laboratory finding is an extremely low or undetectable C-peptide level, confirming absolute endogenous insulin deficiency [71,77,78]. Testing for islet autoantibodies is recommended to support the diagnosis and provide prognostic information but is not essential. Regular blood glucose monitoring at baseline and at each ICI cycle is recommended to facilitate early detection of ICI-DM. For the diagnostic evaluation of new onset hyperglycemia, it is recommended to perform a basic metabolic panel, serum C-peptide and HbA1c [18,79]. These laboratory tests are crucial for the differential diagnosis and help to differentiate from other possible causes such as type 2 diabetes mellitus, stress-induced hyperglycemia or glucocorticoid-induced hyperglycemia.

In cases of DKA, treatment should follow standard protocols, including fluid resuscitation, insulin infusion and electrolyte correction [80]. Due to the irreversible loss of β-cell function, patients require lifelong insulin replacement therapy [65]. The standard of care is basal-bolus therapy, while corticosteroids have no role in treating the underlying autoimmune process of ICI-DM and will exacerbate hyperglycemia [25]. The guidelines recommend maintaining ICI during a severe metabolic event such as DKA [18,79,81]. After the patient’s condition has stabilized with insulin and good glycemic control has been achieved, ICI can usually be resumed. Permanent discontinuation of ICI is not usually necessary solely due to the development of diabetes.

Table 1 provides an overview of studies on endocrine irAEs from ICI therapy.

## 4. Infectious Complications in ICI-Treated Patients

The ICIs significantly improve cancer treatment, achieving increased survival rates and sustained responses in diverse malignant conditions. However, despite their apparent clinical efficacy, recent evidence indicates that ICIs may increase susceptibility to infectious complications through two biologically different but frequently overlapping mechanisms [82]. One mechanism involves direct immune dysregulation, in which excessive T-cell activation paradoxically promotes pathogen persistence and tissue injury. This process, known as ICI therapy-induced dysregulated immunity (ITI-DI), represents a state of hyperinflammatory immune overactivation rather than classical immunodeficiency. It has been described even in patients who have not received corticosteroids or other immunosuppressive drugs [83]. Such dysregulation may lead to reactivation or worsening of latent infections, most notably tuberculosis, cytomegalovirus (CMV), and certain fungal diseases, where an exaggerated Th1-mediated response ultimately benefits the pathogen rather than the host [84]. It is strengthened by the finding that patients with hereditary CTLA-4 dysfunction or mutations demonstrate increased vulnerability to recurrent infections, particularly respiratory infections like tuberculosis [85]. By contrast, the second pathway reflects indirect, iatrogenic immunosuppression, typically resulting from corticosteroids or TNF-α inhibitors used to manage immune-related adverse events (irAEs). These agents decrease protective immune responses, consequently predisposing patients to opportunistic infections such as Pneumocystis jirovecii pneumonia, invasive aspergillosis, and cytomegalovirus reactivation [86]. Clearly distinguishing between these two processes is crucial for clinical decision-making: ITI-DI reflects an overactive, dysregulated immune response requiring modulation and targeted antimicrobial therapy, whereas iatrogenic infections demand mitigation of immunosuppressive burden and preventive prophylaxis.

**Figure 2:**
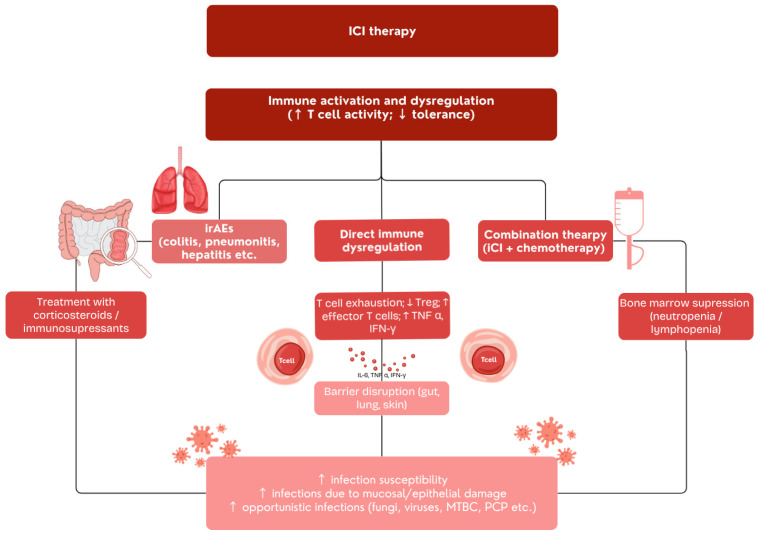
Proposed mechanisms underlying the increased susceptibility to infectious complications during immune checkpoint inhibitor therapy.

Recent evidence supports both mechanisms, emphasizing that ICIs may increase susceptibility to infectious complications through immune dysregulation caused directly by ICI-induced T-cell activation and indirectly through immunosuppressive therapies used to treat irAEs (Figure 2, Table 2 and Table 3). Current clinical observations indicate a rising incidence of bacterial, viral, fungal, and opportunistic infections, including the reactivation of latent infections such as tuberculosis and hepatitis [82,87,88]. According to the European Society of Clinical Microbiology and Infectious Diseases, ICIs themselves do not appear to intrinsically increase infection risk. However, the immunosuppressive therapies used to control immune-related adverse events (irAEs) can predispose patients to secondary or opportunistic infections [89]. Identifying infections that imitate irAEs is particularly important because the therapy is significantly distinct. Incorrectly diagnosing infections can result in delayed diagnosis and treatment, exacerbating the infectious disease due to the administering of corticosteroids and other immunosuppressants to manage the presumed irAEs [85].

### 4.1. Infection Risk from ICI Therapy Alone

The use of ICIs alone does not appear to be associated with a general increase in the overall incidence of infections [89]. For instance, Del Castillo et al. reported that approximately 7% of melanoma patients treated with immune checkpoint inhibitors developed infections [90]. However, their administration may directly predispose certain subgroups of patients to specific infections, such as tuberculosis [91]. T-cell immunity plays a crucial role in defending the body against infections, including tuberculosis, as well as various fungal and viral diseases. ICIs, especially those targeting the PD-1/PD-L1 pathway, are known to restore weakened T-cell responses, which may assist in controlling chronic infections such as HIV or hepatitis. However, this boost in immune activity is not always beneficial—it can sometimes trigger an exaggerated response to previously silent infections, observed in HIV patients who develop immune reconstitution inflammatory syndrome (IRIS) after initiating antiretroviral treatment [84,92]. Further insight into this susceptibility comes from studies of genetic mutations affecting immune checkpoint pathways. Genetic mutations in CTLA-4 and PD-1 receptors suggest that pharmacological inhibition of those pathways may result in infectious complications. For instance, CTLA-4 haploinsufficiency and LRBA deficiency (LATAIE), both characterized by impaired CTLA-4 signaling, are associated with Treg dysfunction, systemic autoimmunity and recurrent infections, especially of the respiratory tract [93,94]. A cohort study of individuals with LATAIE showed that 71% experienced recurrent respiratory and urinary tract infections. The spectrum of bacterial pathogens included *Escherichia coli*, *Klebsiella pneumoniae*, *Haemophilus influenzae*, *Pseudomonas aeruginosa*, *Campylobacter*, and *Staphylococcus aureus*. Reported viral pathogens encompass cytomegalovirus (CMV), adenovirus, norovirus, and varicella zoster virus (VZV), while fungal infections such as *Candida* were also noted [94].

Recent studies revealed that factors beyond corticosteroids and immunosuppression may contribute to the risk of infections following ICI therapy. Various data indicate that hyperinflammatory dysregulated immunity associated with ICIs may promote the development of infections. To distinguish these from infections caused by immunosuppressive therapy, it has been proposed to classify them as ITI-DI. ITI-DI may represent a distinct pathogenic mechanism in which an exaggerated host immune response, resulting from the suppression of immunological checkpoints, paradoxically benefits the pathogen. This mechanism is particularly relevant in infections with critical T-cell-mediated immunity, such as tuberculosis, fungal diseases, and certain viral infections [83]. Two additional mechanisms may be implicated in the development of infectious illnesses related to the use of ICIs. The first concept posits that immunosuppression is associated with immunological checkpoint-related leukopenia/lymphopenia. It was based on observations in the KEYNOTE010 research, which reported the incidence of low-grade lymphopenia of 0.6–1.2% with pembrolizumab, and from the CheckMate017 study, which reported mild to severe-grade leukopenia in 1.0% of patients treated with nivolumab [92]. The second concept suggests a hypersensitive response comparable to IRIS. It was derived from two case reports documenting the occurrence of pulmonary tuberculosis and pericardial tamponade due to hypersensitivity reactions to tuberculosis reactivation in lung cancer patients undergoing nivolumab therapy [92].

Stimulation to the function of T helper-1 (Th1) cells may account for the sporadic recurrence of tuberculosis observed in certain patients treated with PD-1 antibodies [88,95]. Pre-clinical models confirmed this, showing that PD-1-deficient mice exhibit heightened susceptibility to *Mycobacterium tuberculosis* infection [95]. The increased mortality in these animals is due to the enhanced production of IFN-γ by Th1 cells. The observation that the depletion of CD4+ T-cells preserved the mice and the recognized function of Th1 cells in controlling *M. tuberculosis* highlights the necessity of stabilizing T-cell reactivation for pathogen eradication while simultaneously preventing collateral tissue immunopathology. The blockade of the PD-1/PD-L1 pathway may compromise the immune system’s defense against infections, consequently elevating the risk of tuberculosis occurrence, even in individuals not receiving immunosuppressive therapy. Notably, cases of both acute tuberculosis and recurrence of latent tuberculosis have been recorded in patients on PD-1 therapy [96].

Several studies have described reactivation of latent infections, including tuberculosis, CMV, VZV, HBV, and C. difficile, in patients receiving ICI therapy, some even in the absence of additional immunosuppression [85,97,98]. Details are summarized with other relevant studies in Table 4.

Specific symptoms of coronavirus disease 2019 (COVID-19) may resemble ICI-related pneumonitis. Differentiating between the two disorders is essential but complex and may necessitate invasive methods like bronchoscopy. Despite the widespread use of SARS-CoV-2 virus testing prior to the onset of anticancer therapy in numerous oncological centers, there exists a notable incidence (12%) of false-negative results from reverse-transcription PCR assays. Consequently, patients with COVID-19 may be mistakenly perceived as having ICI-related pneumonitis [99].

### 4.2. Infections Associated with Immunosuppressive Treatment of irAEs

The administration of ICIs is frequently accompanied by distinctive irAEs, which arise due to heightened immune activation. irAEs are typically reversible when treated according to standard guidelines utilizing immunosuppressive agents such as steroids or, in cases of resistance, tumor necrosis factor alpha inhibitors (infliximab) [90]. Immunosuppression used to manage irAEs may increase susceptibility to infections. Opportunistic infections such as *Pneumocystis jiroveci* pneumonia, cytomegalovirus-induced hepatitis, and invasive aspergillosis have been reported in several case studies involving melanoma patients treated with the CTLA-4 inhibitor ipilimumab [100]. Recent reports have expanded our understanding of opportunistic infections in patients treated with ICIs. Alongside the more commonly described fungal and viral pathogens, newly reported cases include infections caused by Nocardia species, invasive Candida and Cryptococcus disease, Listeria monocytogenes meningitis, as well as reactivation of Epstein–Barr virus and human herpesvirus 6 (HHV-6). In some instances, these infections have manifested with unusually severe or disseminated courses [101,102]. While most evidence highlights the increased susceptibility to infection, recent data suggest that the immunomodulatory effects of ICIs may also vary depending on the pathogen involved. A population-based study from South Korea found that lung cancer patients treated with immune checkpoint inhibitors had a lower incidence of herpes zoster compared with those who did not receive immunotherapy. This observation suggests that immune modulation induced by ICIs may exert pathogen-specific effects, potentially influencing the risk of viral reactivation [103]. Similarly, a large nationwide cohort reported no significant increase in Pneumocystis jirovecii pneumonia among ICI-treated patients, indicating that ICIs alone may not uniformly heighten the risk of all opportunistic infections [104].

A comprehensive study at Memorial Sloan Kettering Cancer Centre examined 740 melanoma patients in the initial year of ICI therapy to evaluate infection risk. Severe infections were recorded in 7% of patients, with bacterial pathogens responsible for about 80% of instances, predominantly presenting as pneumonia, intra-abdominal, or bloodstream infections. Several viral and fungal infections were also reported. The majority of affected individuals had been administered corticosteroids, indicating that immunosuppressive therapy for irAEs may be a significant factor [90]. A study of patients with non-small cell lung cancer treated with the PD-1 inhibitor nivolumab revealed an infection rate of 19%, with respiratory tract infections being the most common. These were typically caused by community-acquired bacteria such as *Streptococcus pneumoniae*, *Haemophilus influenzae*, *Staphylococcus aureus*, or viruses like Influenza [92]. Corticosteroids were used to manage irAEs in about 50% of patients, though without statistical significance [92]. Other studies similarly link corticosteroid use during ICI therapy to higher risk of severe infections, most often bacterial—primarily urinary tract, followed by pneumonia and skin/soft-tissue infections [105].

Moreover, the administration of alternative immunosuppressive drugs for managing severe irAEs may elevate the risk of opportunistic infections and require additional clinical attention [106]. Furthermore, CMV infections have been reported in individuals with ICI-induced colitis who received corticosteroids and infliximab, categorized as refractory cases [107]. A recent systematic review encompassing over 1000 cancer patients found that patients administered immunosuppressants for gastrointestinal and hepatobiliary irAEs encountered more adverse events, with an overall infection rate of 22.3% [106]. The potential of ICI therapy to predispose individuals to invasive fungal infections is a matter of clinical interest. Infections of this nature are rare with the administration of ICI, and the majority of cases documented in the existing literature have been assigned to invasive aspergillosis and pneumocystis pneumonia [90,92]. Uchida et al. described a lung adenocarcinoma patient whose CPPA worsened under nivolumab despite near-complete tumor response, likely due to IRIS-like immune hyperactivation rather than immunosuppression [108].

### 4.3. Endocrine Dysfunction as a Risk Factor for Infections

While endocrine complications are well-documented as irAEs, their potential role in predisposing patients to infections remains underexplored. Current evidence does not support a direct causal relationship between endocrine irAEs and viral or bacterial infections, including HIV, hepatitis B/C, or tuberculosis. However, overlapping clinical presentations and secondary factors, such as corticosteroid therapy or metabolic disturbances, may increase susceptibility to infection or complicate diagnosis. Therefore, clinicians should stay mindful of these potential interactions, though direct evidence remains limited.

#### 4.3.1. Thyroid Dysfunction and Infection Risk

The ICI-induced thyroid dysfunctions, including hypothyroidism, hyperthyroidism, and thyroiditis, are among the most common endocrine irAEs. However, direct evidence linking thyroid dysfunction resulting from ICI therapy to an increased infection risk is limited [109]. Data from non-ICI settings suggest that hypothyroidism, particularly when inadequately treated, may be associated with elevated susceptibility to infections. A nationwide Taiwanese cohort study demonstrated a 38% higher risk of pneumonia in patients with newly diagnosed hypothyroidism compared to controls, while thyroid replacement therapy significantly reduced this risk [110]. Likewise, the Norwegian HUNT2 study reported a 20–30% increased risk of bloodstream infections in patients with thyroid dysfunction [111]. Hypothyroidism has been associated not only with higher incidence of systemic infections, but also with localized infections. In joint arthroplasty patients, hypothyroidism was linked to higher periprosthetic infection rates (3.4% vs. 1.4%) [112], likely due to impaired immunity. This suggests that unrecognized thyroid dysfunction during ICI therapy may raise infection risk, emphasizing proactive endocrine monitoring.

#### 4.3.2. Hypophysitis and Adrenal Insufficiency

Hypophysitis does not directly increase infection risk, but secondary adrenal insufficiency as a result of hypophysitis can lead to impaired immune function. Adrenal insufficiency, due to low cortisol production, puts patients at a bigger risk of severe infections and poor outcomes. Evidence on infection risk specifically in immune checkpoint inhibitor (ICI)–induced adrenal insufficiency is currently lacking. Available data largely derive from non-ICI populations. Infections are the third leading cause of mortality in patients with adrenal insufficiency, with mortality risk highest within the first year after diagnosis [113]. Furthermore, infections are the second most common cause of hospital admission in this population [114]. A Swedish study further emphasized these risks, showing that patients with adrenal insufficiency and COVID 19 infection have more than twice the risk of hospitalization, ICU admission, and death [115]. However, it focused on infection-related mortality in adrenal insufficiency broadly, not specifically on endocrine irAEs or cancer patients treated with immune checkpoint inhibitors. Therefore, while it is biologically plausible that cortisol deficiency during ICI therapy could increase susceptibility to severe infections, this relationship remains theoretical and requires further study.

#### 4.3.3. Diabetes Mellitus and Infection Susceptibility

Although data specific to infection risk in ICI-DM are limited, substantial evidence from studies of T1DM in general populations demonstrates a strong association between hyperglycemia and increased infection susceptibility. Patients with T1DM exhibit both innate and adaptive immune dysfunction, impairing host defenses against various bacterial, fungal, and mycobacterial pathogens, particularly *Candida albicans*, *Staphylococcus aureus*, and *Mycobacterium tuberculosis* [116]. Mendelian randomization suggests a genetic predisposition to infections in T1DM, though glycemic control remains the key modifiable factor [117]. Prospective studies confirm that diabetes, especially with poor control, increases risk of respiratory, urinary, skin/soft tissue, and fungal infections, with severe outcomes at HbA1c ≥ 11% [118,119]. In ICI-DM, abrupt glycemic fluctuations and corticosteroid use may heighten infection risk, underscoring the need for strict glucose monitoring, early insulin initiation, and close infection surveillance.

Table 4 provides an overview of studies reporting infections during ICI therapy.

**Table 4:** Studies on infections associated with immune checkpoint inhibitor therapy.

Author, Year (Journal)	Study Design & Population	Infection Focus	Key Results/Findings
Del Castillo et al., 2016 (Clin. Infect. Dis.) [90]	Retrospective analysis: melanoma patients treated with ICIs	General infection risk	Approximately 7% developed infections; mostly bacterial (85%), some opportunistic cases.
Zhang et al., 2019 (J. Immunother. Cancer) [97]	114 HBsAg+ cancer patients treated with PD-1/PD-L1 inhibitors	HBV reactivation	5.3% experienced HBV reactivation (median 18 weeks), 5 developed hepatitis; prophylaxis reduced risk significantly.
Uchida et al., 2018 (Respirol. Case Rep.) [108]	Single patient, nivolumab therapy	Fungal infection (chronic pulmonary aspergillosis)	Nearly complete tumor response but worsening fungal infection; immune hyperactivation suspected (IRIS-like).
Picchi et al., 2018 (Clin. Microbiol. Infect.) [88] Fujita et al., 2019 (Respir. Med.) [92] Langan et al., 2020 (Lancet Oncol.) [96]	Multiple reports, mostly melanoma and NSCLC patients	Tuberculosis reactivation under PD-1 therapy	Several cases of active/latent TB reactivation; attributed to PD-1 pathway blockade and Th1 hyperactivation.
Franklin et al., 2017 (Eur. J. Cancer) [107]	Melanoma patients with ICI-colitis treated with steroids/infliximab	CMV colitis/hepatitis	CMV reactivation mimicking refractory autoimmune colitis; confirmed by biopsy, mostly responsive to ganciclovir.
Picasso et al., 2023 (Radiol. Med.) [99]	Cancer patients on ICIs during pandemic	Differentiating ICI-pneumonitis from COVID-19	False-negative RT-PCR (estimated at 12%); some COVID-19 cases misdiagnosed as ICI pneumonitis.
Babacan and Tanvetyanon, 2019. (J. Immunother.) [98]	Mostly lung adenocarcinoma patients with ICI-colitis	*Clostridioides difficile* infection	Diarrhea exacerbation in ICI colitis warrants suspicion for *Clostridioides difficile* infection
Gudiol et al., 2022 (Open Forum Infect. Dis.) [85]	Mostly melanoma, lung adenocarcinoma and NSCLC	VZV skin and CNS infections, vasculopathy + other pathogens	Assessment strategies to exclude occult infection in irAEs should follow a syndromic, suspicion-driven approach

ICI—immune checkpoint inhibitor; NSCLC—non-small cell lung cancer; HBsAg—hepatitis B surface antigen; PD-1—programmed cell death protein-1; PD-L1—programmed death-ligand 1; HBV—hepatitis B virus; IRIS—immune reconstitution inflammatory syndrome; TB—tuberculosis; Th1—T helper cell type 1; CMV—cytomegalovirus; RT-PCR—reverse transcription polymerase chain reaction; VZV—varicella zoster virus; CNS—central nervous system.

## 5. Challenges, Controversies, and Future Direction

### 5.1. Potential Challenges in the Differential Diagnosis of irAEs and Infectious Complications Associated with Checkpoint Inhibitors

Given that the number of oncology patients is increasing and that checkpoint inhibitors are used in more and more indications and types of tumors, from neoadjuvant to metastatic treatment, the question arises whether the guidelines for monitoring, diagnosing and treating the adverse events of this therapy are sufficiently precise. Treatment is mainly focused on corticosteroid and hormone replacement therapy, but given that these drugs will be used by a large number of patients for a long time, it is necessary to find better and more precise ways to predict and treat certain adverse events. Also, given the growing number of oncology patients, oncology clinics are often overcrowded, and it is possible that doctors do not have time to analyze the patient’s symptoms in detail. This can be especially disastrous if infections associated with checkpoint inhibitors occur that remain unrecognized. Therefore, for this aspect, there should be quality algorithms for treatment and diagnosis in the event of the appearance of certain symptoms.

### 5.2. Future Approach to the Treatment of irAEs

In addition to the aforementioned biomarkers that could predict which patients will develop irAEs, a number of new drugs could also contribute to the effectiveness of treatment. Rituximab, a drug widely used in hematological malignancies, has been shown to be useful in the treatment of neurotoxicity (e.g., myasthenia gravis) and nephrotoxicity caused by ICIs [11]. The use of inhibitors of various cytokines in the treatment and prophylaxis of irAEs is also being actively investigated. Of particular importance is interleukin-6, which has a significant proinflammatory effect in these adverse events, and its inhibitor tocilizumab. Tocilizumab is already used in the treatment of rheumatoid arthritis and giant cell arteritis [11]. In a study of 2 melanoma treatment centers, 20 patients with irAEs were treated with tocilizumab, in addition to 2 patients with existing autoimmune diseases who received it prophylactically [12]. It has been shown to be successful in treating adverse events while reducing CRP and in preventing the exacerbation of autoimmune diseases. Also, according to an analysis in which 91 patients received tocilizumab for the treatment of adverse events, 85% of patients were successfully treated and did not experience disease progression [120]. Other cytokine inhibitors are also being investigated, such as the IL-17 inhibitors bimekizumab and secukinumab and the IL-23 inhibitor guselkumab [11].

These drugs could significantly reduce the use of corticosteroids in the treatment of irAEs, which would potentially lead to a reduction in opportunistic infections caused by corticosteroids. It is also possible that they could reduce ICI therapy-induced dysregulated immunity, which also favors infections. However, many more clinical trials for this indication are needed because of the possible collision with the antitumor activity of ICI and consequently a reduction in their efficacy.

What is encouraging is the apparent increase in global scientific interest in this field, as evidenced by the substantial rise in studies published in recent years on IrAEs, as well as the efficacy and safety of ICIs [121]. This growing body of research indicates that both investigators and clinicians have progressively come to appreciate the clinical significance of IrAEs and the critical importance of ensuring the safest and most responsible use of ICIs in practice.

## 6. Conclusions

ICIs have transformed cancer therapy by reactivating T cell-mediated anti-tumor immunity, but this potent immune stimulation can also cause a wide range of irAEs. Among the most common are endocrine dysfunctions, including thyroiditis, hypophysitis, which can lead to permanent secondary adrenal insufficiency, and a rare but severe form of autoimmune DM. At the same time, infectious complications observed in ICI-treated patients appear to be influenced primarily by the immunosuppressive therapies used to manage irAEs, as well as by the immune consequences of the endocrine dysfunctions themselves. Evidence also suggests that these endocrine irAEs, particularly adrenal insufficiency and diabetes, may further impair immune function and predispose patients to a significantly higher risk of infection. Therefore, greater interdisciplinary awareness is crucial for the early recognition and treatment of these complex endocrine and infectious complications, which is critical for improving the safety and outcomes of patients receiving immunotherapy.

## Figures and Tables

**Table 1:** Studies on endocrine disturbances from immune checkpoint inhibitor therapy.

Author, Year (Journal)	Study Design & Population	Endocrine Focus	Key Results/Findings
Barroso-Sousa et al., 2018 (JAMA Oncol.) [35]	Systematic review & meta-analysis; 38 clinical trials	All endocrine irAEs	Incidence highest with combination therapy; thyroid dysfunction most common, followed by hypophysitis and adrenal insufficiency.
De Filette et al., 2019 (Horm. Metab. Res.) [30]	Systematic review & meta-analysis	Endocrine irAEs overall	Higher risk of thyroid dysfunction with PD-1/PD-L1 inhibitors; hypophysitis strongly associated with CTLA-4 inhibitors.
Min et al., 2015 (Clin. Cancer Res.) [48]	Retrospective cohort; ipilimumab-treated patients	Hypophysitis	High-dose corticosteroids did not improve pituitary recovery; most patients required lifelong hormone replacement.
Di Dalmazi et al., 2019 (Expert. Rev. Endocrinol. Metab.) [42]	10-year assessment review	Hypophysitis	Median onset 2–4 months; CTLA-4 inhibitors major driver; secondary adrenal insufficiency often permanent.
Cui et al., 2022 (Ann. Transl. Med.) [58]	Large-sample case series	Adrenal insufficiency	Highlighted clinical features and frequency of adrenal insufficiency with ICIs; PD-1/PD-L1 > CTLA-4.
Grouthier et al., 2020 (Oncologist) [52]	WHO VigiBase pharmacovigilance study	Primary adrenal insufficiency	Rare but potentially life-threatening; emphasized need for early recognition.
Akturk et al., 2019 (Diabet. Med.) [66]	Systematic review & meta-analysis; 71 cases	ICI-induced T1DM	Incidence 0.2–1.9%; often acute onset, presenting with DKA; mostly with PD-1/PD-L1 inhibitors.
Wu et al., 2023 (Diabetes Care) [72]	Systematic review; 192 cases	Checkpoint inhibitor–associated DM	Distinct phenotype from classic T1DM; abrupt presentation, low antibody positivity, severe hyperglycemia.
Baden et al., 2019 (Diabetol. Int.) [60]	Multicenter cohort; Japan	Anti-PD-1 therapy diabetes	Characterized rapid-onset ICI-DM; high rates of ketoacidosis.
Tsang et al., 2019 (J. Clin. Endocrinol. Metab.) [61]	Clinical cohort	ICI-associated autoimmune diabetes	Distinct course vs. T1DM; often antibody-negative, aggressive course.
Yamauchi I., Yabe D., 2025 (Eur. Thyroid. J.) [28]	Clinical review	Thyroid dysfunction	ICI-thyroid dysfunction frequent; PD-1/PD-L1 linked to hypothyroidism, CTLA-4 to thyroiditis.
Chalan et al., 2018 (J. Endocrinol. Investig.) [29]	Narrative review	Thyroid dysfunction	Thyroiditis followed by hypothyroidism common; permanent dysfunction in many cases.

ICI—immune checkpoint inhibitor; CTLA-4—cytotoxic T-lymphocyte–associated protein 4; PD-1/PD-L1—programmed cell death protein 1/programmed death-ligand 1; irAEs—immune-related adverse events; T1DM—type 1 diabetes mellitus; DKA—diabetic ketoacidosis.

**Table 2:** Mechanistic infection risks associated with immune checkpoint inhibitor (ICI) therapy.

	Predisposing Event	Potential Mechanism	Potential Pathogens/Infections
Infection risk from ICI therapy alone	Direct immune dysregulation by ICIs	Loss of checkpoint control, T-cell hyperactivation, IRIS-like phenomena	*Mycobacterium tuberculosis* (new or reactivation); Fungal infections (*Candida* spp., *Aspergillus* spp.); Viral reactivations (HIV IRIS, CMV colitis/gastritis, VZV CNS disease, HSV family viruses); HBV reactivation; SARS-CoV-2 (COVID-19, differential with ICI-pneumonitis); *Clostridioides difficile* (CDAD, even without antibiotics)
	Genetic predisposition	CTLA-4 haploinsufficiency, LRBA deficiency, impaired Treg function	Recurrent respiratory & urinary tract infections: *Escherichia coli*, *Klebsiella pneumoniae*, *Haemophilus influenzae*, *Pseudomonas aeruginosa*, *Campylobacter* spp., *Staphylococcus aureus*; Viral: CMV, adenovirus, norovirus, VZV; Fungal: *Candida* spp.
	ICI-related cytopenia	Leukopenia/lymphopenia observed with pembrolizumab, nivolumab	General increased risk of bacterial (respiratory, urinary tract) infections, viral reactivations, opportunistic fungi

ICI—immune checkpoint inhibitor; IRIS—immune reconstitution inflammatory syndrome; CTLA-4—cytotoxic T-lymphocyte–associated protein 4; LRBA—lipopolysaccharide-responsive and beige-like anchor protein; Treg—regulatory T-cell; PD-1/PD-L1—programmed cell death protein 1/programmed death-ligand 1; CMV—cytomegalovirus; VZV—varicella-zoster virus; HSV—herpes simplex virus; HBV—hepatitis B virus; COVID-19—coronavirus disease 2019; CDAD—*Clostridioides difficile*–associated disease.

**Table 3:** Secondary infection risks associated with the management of immune-related adverse events (irAEs) and endocrine dysfunction.

	Predisposing Event	Potential Mechanism	Potential Pathogens/Infections
Infections associated with immunosuppressive treatment of irAEs	Corticosteroids, TNF-α inhibitors (e.g., infliximab) therapy	Immunosuppression	Bacterial: *Streptococcus pneumoniae*, *Haemophilus influenzae*, *Staphylococcus aureus* (pneumonia, bloodstream, intra-abdominal, urinary tract, SSTI); Viral: Influenza, CMV hepatitis/colitis; Fungal: *Pneumocystis jirovecii* pneumonia, invasive aspergillosis
Endocrine dysfunction as a risk factor for infections	Thyroid disorders	Hypothyroidism, hyperthyroidism, thyroiditis	Increased risk of pneumonia, bloodstream infections, localized infections (e.g., periprosthetic joint infection); broad bacterial spectrum
	Hypophysitis with adrenal insufficiency	Low cortisol → impaired immune function	Severe bacterial and viral infections (incl. higher COVID-19 mortality); sepsis, hospitalization, infection-related mortality
	ICI-induced diabetes mellitus	Hyperglycemia, immune dysfunction, abrupt glycemic fluctuations	Bacterial: respiratory, urinary tract, skin/soft tissue; *Staphylococcus aureus*; Mycobacterial: *Mycobacterium tuberculosis*; Fungal: *Candida albicans*; Risk amplified with poor glycemic control

ICI—immune checkpoint inhibitor; irAEs—immune-related adverse events; TNF-α—tumor necrosis factor alpha; CMV—cytomegalovirus; COVID-19—coronavirus disease 2019; SSTI—skin and soft tissue infection.

## Data Availability

No new data were created or analyzed in this study. Data sharing is not applicable to this article.

## Abbreviations

The following abbreviations are used in this manuscript:

ACTH	Adrenocorticotropic hormone
AI	Adrenal insufficiency
CDAD	*C. difficile* associated disease
CMV CNS	Cytomegalovirus Central nervous system
COVID-19	Coronavirus disease 2019
CPPA	Chronic progressive pulmonary aspergillosis
CRP	C-reactive protein
CT	Computed tomography
CTLA-4	Cytotoxic T-lymphocyte-associated protein 4
DKA	Diabetic ketoacidosis
DM	Diabetes mellitus
fT4	Free thyroxine
HbA1c	Glycated hemoglobin
HBV	Hepatitis B virus
HLA	Human leukocyte antigen
ICI	Immune checkpoint inhibitor
ICI-DM	Immune checkpoint inhibitor-associated diabetes mellitus
IFN-γ	Interferon-gamma
IL	Interleukin
irAEs	Immune-related adverse events
IRIS	Immune reconstitution inflammatory syndrome
ITI-DI	ICI therapy-induced dysregulated immunity
MRI	Magnetic resonance imaging
PCR	Polymerase chain reaction
PD-1	Programmed cell death protein 1
PD-L1	Programmed death-ligand 1
T1DM	Type 1 diabetes mellitus
Treg	Regulatory T-lymphocytes
TSH	Thyroid-stimulating hormone
VZV	Varicella-zoster virus

## References

[1] Shiravand Y., Khodadadi F., Kashani S.M.A., Hosseini-Fard S.R., Hosseini S., Sadeghirad H., Ladwa R., O’Byrne K., Kulasinghe A. (2022). Immune Checkpoint Inhibitors in Cancer Therapy. Curr. Oncol..

[2] Haanen J.B.A.G., Robert C. (2015). Immune Checkpoint Inhibitors. Prog. Tumor Res..

[3] Hargadon K.M., Johnson C.E., Williams C.J. (2018). Immune checkpoint blockade therapy for cancer: An overview of FDA-approved immune checkpoint inhibitors. Int. Immunopharmacol..

[4] Naimi A., Mohammed R.N., Raji A., Chupradit S., Yumashev A.V., Suksatan W., Shalaby M.N., Thangavelu L., Kamrava S., Shomali N. (2022). Tumor immunotherapies by immune checkpoint inhibitors (ICIs); the pros and cons. Cell Commun. Signal..

[5] Wolchok J.D., Chiarion-Sileni V., Gonzalez R., Grob J.J., Rutkowski P., Lao C.D., Cowey C.L., Schadendorf D., Wagstaff J., Dummer R. (2022). Long-Term Outcomes with Nivolumab Plus Ipilimumab or Nivolumab Alone Versus Ipilimumab in Patients With Advanced Melanoma. J. Clin. Oncol..

[6] Johnson M.L., Cho B.C., Luft A., Alatorre-Alexander J., Geater S.L., Laktionov K., Kim S., Ursol G., Hussein M., Lim F.L. (2023). Durvalumab with or Without Tremelimumab in Combination with Chemotherapy as First-Line Therapy for Metastatic Non-Small-Cell Lung Cancer: The Phase III POSEIDON Study. J. Clin. Oncol..

[7] Sangro B., Chan S.L., Kelley R.K., Lau G., Kudo M., Sukeepaisarnjaroen W., Yarchoan M., De Toni E.N., Furuse J., Kang Y.K. (2024). Four-year overall survival update from the phase III HIMALAYA study of tremelimumab plus durvalumab in unresectable hepatocellular carcinoma. Ann. Oncol..

[8] Okazaki T., Honjo T. (2006). The PD-1-PD-L pathway in immunological tolerance. Trends Immunol..

[9] Nagasaki J., Ishino T., Togashi Y. (2022). Mechanisms of resistance to immune checkpoint inhibitors. Cancer Sci..

[10] Lentz R.W., Colton M.D., Mitra S.S., Messersmith W.A. (2021). Innate immune checkpoint inhibitors: The next breakthrough in medical oncology?. Mol. Cancer Ther..

[11] Yin Q., Wu L., Han L., Zheng X., Tong R., Li L., Bai L., Bian Y. (2023). Immune-related adverse events of immune checkpoint inhibitors: A review. Front. Immunol..

[12] Dimitriou F., Hogan S., Menzies A.M., Dummer R., Long G.V. (2021). Interleukin-6 blockade for prophylaxis and management of immune-related adverse events in cancer immunotherapy. Eur. J. Cancer.

[13] Lozano A.X., Chaudhuri A.A., Nene A., Bacchiocchi A., Earland N., Vesely M.D., Usmai A., Turner B.E., Steen C.B., Luca B.A. (2022). T cell characteristics associated with toxicity to immune checkpoint blockade in patients with melanoma. Nat. Med..

[14] National Comprehensive Cancer Network NCCN Clinical Practice Guidelines in Oncology (NCCN Guidelines^®^): Management of Immune Checkpoint Inhibitor-Related Toxicities. Version 1.2026. 23 October 2025..

[15] Darnell E.P., Mooradian M.J., Baruch E.N., Yilmaz M., Reynolds K.L. (2020). Immune-Related Adverse Events (irAEs): Diagnosis, Management, and Clinical Pearls. Curr. Oncol. Rep..

[16] Postow M.A., Sidlow R., Hellmann M.D. (2018). Immune-Related Adverse Events Associated with Immune Checkpoint Blockade. N. Engl. J. Med..

[17] Puzanov I., Diab A., Abdallah K., Bingham C.O., Brogdon C., Dadu R., Hamad L., Kim S., Lacouture M.E., LeBoeuf N.R. (2017). Managing toxicities associated with immune checkpoint inhibitors: Consensus recommendations from the Society for Immunotherapy of Cancer (SITC) Toxicity Management Working Group. J. Immunother. Cancer.

[18] Schneider B.J., Naidoo J., Santomasso B.D., Lacchetti C., Adkins S., Anadkat M., Atkins M.B., Brassil K.J., Caterino J.M., Chau I. (2021). Management of Immune-Related Adverse Events in Patients Treated with Immune Checkpoint Inhibitor Therapy: ASCO Guideline Update. J. Clin. Oncol..

[19] Haanen J., Obeid M., Spain L., Carbonnel F., Wang Y., Robert C., Lyon A.R., Wick W., Kostine M., Peters S. (2022). Management of toxicities from immunotherapy: ESMO Clinical Practice Guideline for diagnosis, treatment and follow-up. Ann. Oncol..

[20] Hussaini S., Chehade R., Boldt R.G., Raphael J., Blanchette P., Maleki Vareki S., Fernandes R. (2021). Association between immune-related side effects and efficacy and benefit of immune checkpoint inhibitors—A systematic review and meta-analysis. Cancer Treat. Rev..

[21] Anpalakhan S., Huddar P., Behrouzi R., Signori A., Cave J., Comins C., Cortellini A., Addeo A., Escriu C., McKenuie H. (2023). Immunotherapy-related adverse events in real-world patients with advanced non-small cell lung cancer on chemoimmunotherapy: A Spinnaker study sub-analysis. Front. Oncol..

[22] Peng L., Wang Y., Liu F., Qiu X., Zhang X., Fang C., Qia X., Li Y. (2020). Peripheral blood markers predictive of outcome and immune-related adverse events in advanced non-small cell lung cancer treated with PD-1 inhibitors. Cancer Immunol. Immunother..

[23] Chennamadhavuni A., Abushahin L., Jin N., Presley C.J., Manne A. (2022). Risk Factors and Biomarkers for Immune-Related Adverse Events: A Practical Guide to Identifying High-Risk Patients and Rechallenging Immune Checkpoint Inhibitors. Front. Immunol..

[24] Zhan L., Feng H.F., Liu H.Q., Guo L.T., Chen C., Yao X.L., Sun S.R. (2021). Immune Checkpoint Inhibitors-Related Thyroid Dysfunction: Epidemiology, Clinical Presentation, Possible Pathogenesis, and Management. Front. Endocrinol..

[25] Husebye E.S., Castinetti F., Criseno S., Curigliano G., Decallonne B., Fleseriu M., Higham C.E., Lupi I., Paschou S.A., Toth M. (2022). Endocrine-related adverse conditions in patients receiving immune checkpoint inhibition: An ESE clinical practice guideline. Eur. J. Endocrinol..

[26] Bai X., Chen X., Wu X., Huang Y., Zhuang Y., Lin X. (2019). Immune checkpoint inhibitor-associated thyroid dysfunction: A disproportionality analysis using the WHO adverse drug reaction database, VigiBase. Eur. J. Endocrinol..

[27] Kethireddy N., Thomas S., Bindal P., Shukla P., Hegde U. (2021). Multiple autoimmune side effects of immune checkpoint inhibitors in a patient with metastatic melanoma receiving pembrolizumab. J. Oncol. Pharm. Pract..

[28] Yamauchi I., Yabe D. (2025). Best practices in the management of thyroid dysfunction induced by immune checkpoint inhibitors. Eur. Thyroid. J..

[29] Chalan P., Di Dalmazi G., Pani F., De Remigis A., Corsello A., Caturegli P. (2018). Thyroid dysfunctions secondary to cancer immunotherapy. J. Endocrinol. Investig..

[30] De Filette J., Andreescu C.E., Cools F., Bravenboer B., Velkeniers B. (2019). A Systematic Review and Meta-Analysis of Endocrine-Related Adverse Events Associated with Immune Checkpoint Inhibitors. Horm. Metab. Res..

[31] Sabbagh REl Azar N.S., Eid A.A., Azar S.T. (2020). Thyroid Dysfunctions Due to Immune Checkpoint Inhibitors: A Review. Int. J. Gen. Med..

[32] Karaviti D., Kani E.R., Karaviti E., Gerontiti E., Michalopoulou O., Stefanaki K., Kazakou P., Vasileiou V., Psaltopoulou T., Paschou S.A. (2024). Thyroid disorders induced by immune checkpoint inhibitors. Endocrine.

[33] Singh N., Hocking A.M., Buckner J.H. (2023). Immune related adverse events after immune check point inhibitors: Understanding the intersection with autoimmunity. Immunol. Rev..

[34] Larkin J., Chiarion-Sileni V., Gonzalez R., Grob J.J., Cowey C.L., Lao C.D., Schadendorf D., Dummer R., Smylie M., Rutkowski P. (2015). Combined Nivolumab and Ipilimumab or Monotherapy in Untreated Melanoma. N. Engl. J. Med..

[35] Barroso-Sousa R., Barry W.T., Garrido-Castro A.C., Hodi F.S., Min L., Krop I.E., Tolaney S.M. (2018). Incidence of endocrine dysfunction following the use of different immune checkpoint inhibitor regimens a systematic review and meta-analysis. JAMA Oncol..

[36] El Osta B., Hu F., Sadek R., Chintalapally R., Tang S.C. (2017). Not all immune-checkpoint inhibitors are created equal: Meta-analysis and systematic review of immune-related adverse events in cancer trials. Crit. Rev. Oncol. Hematol..

[37] Joshi M.N., Whitelaw B.C., Palomar M.T.P., Wu Y., Carroll P.V. (2016). Immune checkpoint inhibitor-related hypophysitis and endocrine dysfunction: Clinical review. Clin. Endocrinol..

[38] Jacques J.P., Valadares L.P., Moura A.C., Oliveira M.R.F., Naves L.A. (2023). Frequency and clinical characteristics of hypophysitis and hypopituitarism in patients undergoing immunotherapy—A systematic review. Front. Endocrinol..

[39] Iwama S., De Remigis A., Callahan M.K., Slovin S.F., Wolchok J.D., Caturegli P. (2014). Pituitary expression of CTLA-4 mediates hypophysitis secondary to administration of CTLA-4 blocking antibody. Sci. Transl. Med..

[40] Kanie K., Iguchi G., Bando H., Urai S., Shichi H., Fujita Y., Matsumoto R., Suda K., Yamamoto M., Fukuoka H. (2021). Mechanistic insights into immune checkpoint inhibitor-related hypophysitis: A form of paraneoplastic syndrome. Cancer Immunol. Immunother..

[41] van der Leij S., Suijkerbuijk K.P.M., van den Broek M.F.M., Valk G.D., Dankbaar J.W., van Santen H.M. (2024). Differences in checkpoint-inhibitor-induced hypophysitis: Mono- versus combination therapy induced hypophysitis. Front. Endocrinol..

[42] Di Dalmazi G., Ippolito S., Lupi I., Caturegli P. (2019). Hypophysitis induced by immune checkpoint inhibitors: A 10-year assessment. Expert. Rev. Endocrinol. Metab..

[43] Nguyen H., Shah K., Waguespack S.G., Hu M.I., Habra M.A., Cabanillas M.E., Busaidy N.L., Bassett R., Zhou S., Iyer P.C. (2021). Immune checkpoint inhibitor related hypophysitis: Diagnostic criteria and recovery patterns. Endocr. Relat. Cancer.

[44] Elshafie O., Khalil A.B., Salman B., Atabani A., Al-Sayegh H. (2024). Immune Checkpoint Inhibitors-Induced Endocrinopathies: Assessment, Management and Monitoring in a Comprehensive Cancer Centre. Endocrinol. Diabetes Metab..

[45] Sang H., Cho Y.K., Go S.H., Kim H.J., Koh E.H. (2024). Patterns of hormonal changes in hypophysitis by immune checkpoint inhibitor. Korean J. Intern. Med..

[46] Higham C.E., Olsson-Brown A., Carroll P., Cooksley T., Larkin J., Lorigan P., Morganstein D., Trainer P.J. (2018). Society for endocrinology endocrine emergency guidance: Acute management of the endocrine complications of checkpoint inhibitor therapy. Endocr. Connect..

[47] Wright J.J., Powers A.C., Johnson D.B. (2021). Endocrine Toxicities of Immune Checkpoint Inhibitors. Nat. Rev. Endocrinol..

[48] Min L., Hodi F.S., Giobbie-Hurder A., Ott P.A., Luke J.J., Donahue H., Davis M., Carroll R.S., Kaiser U.B. (2015). Systemic high-dose corticosteroid treatment does not improve the outcome of ipilimumab-related hypophysitis: A retrospective cohort study. Clin. Cancer Res..

[49] Martin-Grace J., Dineen R., Sherlock M., Thompson C.J. (2020). Adrenal insufficiency: Physiology, clinical presentation and diagnostic challenges. Clin. Chim. Acta.

[50] Martella S., Lucas M., Porcu M., Perra L., Denaro N., Pretta A., Deias G., Willard-Gallo K., Parra H.S., Saba L. (2023). Primary adrenal insufficiency induced by immune checkpoint inhibitors: Biological, clinical, and radiological aspects. Semin. Oncol..

[51] Cukier P., Santini F.C., Scaranti M., Hoff A.O. (2017). Endocrine side effects of cancer immunotherapy. Endocr. Relat. Cancer.

[52] Grouthier V., Lebrun-Vignes B., Moey M., Johnson D.B., Moslehi J.J., Salem J.E., Bachelot A. (2020). Immune Checkpoint Inhibitor--Associated Primary Adrenal Insufficiency: WHO VigiBase Report Analysis. Oncologist.

[53] Husebye E.S., Pearce S.H., Krone N.P., Kämpe O. (2021). Adrenal insufficiency. Lancet.

[54] Helderman N.C., Lucas M.W., Blank C.U. (2023). Autoantibodies involved in primary and secondary adrenal insufficiency following treatment with immune checkpoint inhibitors. Immuno-Oncol. Technol..

[55] Lanzolla G., Coppelli A., Cosottini M., Del Prato S., Marcocci C., Lupi I. (2019). Immune Checkpoint Blockade Anti–PD-L1 as a Trigger for Autoimmune Polyendocrine Syndrome. J. Endocr. Soc..

[56] Lewis A., Thant A.A., Aslam A., Aung P.P.M., Azmi S. (2024). Diagnosis and management of adrenal insufficiency. Clin. Med..

[57] Bornstein S.R., Allolio B., Arlt W., Barthel A., Don-Wauchope A., Hammer G.D., Husebye E.S., Merke D.P., Murad M.H., Stratakis C.A. (2015). Diagnosis and Treatment of Primary Adrenal Insufficiency: An Endocrine Society Clinical Practice Guideline. J. Clin. Endocrinol. Metab..

[58] Cui K., Wang Z., Zhang Q., Zhang X. (2022). Immune checkpoint inhibitors and adrenal insufficiency: A large-sample case series study. Ann. Transl. Med..

[59] Mitrache M.L., Reghina A.D., Stoian I.S., Fica S. (2025). Immune Checkpoint Inhibitor-Induced Diabetes Mellitus-A Brief Review and Three Case Reports. J. Clin. Med..

[60] Baden M.Y., Imagawa A., Abiru N., Awata T., Ikegami H., Uchigata Y., Oikawa Y., Osawa H., Kajio H., Kawasaki E. (2019). Characteristics and clinical course of type 1 diabetes mellitus related to anti-programmed cell death-1 therapy. Diabetol. Int..

[61] Tsang V.H.M., McGrath R.T., Clifton-Bligh R.J., Scolyer R.A., Jakrot V., yama Guminski A.D., Long G.V., Menzies A.M. (2019). Checkpoint Inhibitor-Associated Autoimmune Diabetes Is Distinct from Type 1 Diabetes. J. Clin. Endocrinol. Metab..

[62] Kani E.R., Karaviti E., Karaviti D., Gerontiti E., Paschou I.A., Saltiki K., Stefanaki K., Psaltopoulou T., Paschou S.A. (2025). Pathophysiology, diagnosis, and management of immune checkpoint inhibitor-induced diabetes mellitus. Endocrine.

[63] Quandt Z., Perdigoto A., Anderson M.S., Herold K.C. (2025). Checkpoint Inhibitor-Induced Autoimmune Diabetes: An Autoinflammatory Disease. Cold Spring Harb. Perspect. Med..

[64] Wright J.J., Salem J.E., Johnson D.B., Lebrun-Vignes B., Stamatouli A., Thomas J.W., Herold K.C., Moslehi J., Powers A.C. (2018). Increased reporting of immune checkpoint inhibitor-associated diabetes. Diabetes Care.

[65] Kotwal A., Haddox C., Block M., Kudva Y.C. (2019). Immune checkpoint inhibitors: An emerging cause of insulin-dependent diabetes. BMJ Open Diabetes Res. Care.

[66] Akturk H.K., Kahramangil D., Sarwal A., Hoffecker L., Murad M.H., Michels A.W. (2019). Immune checkpoint inhibitor-induced Type 1 diabetes: A systematic review and meta-analysis. Diabet. Med..

[67] Ansari M.J.I., Salama A.D., Chitnis T., Smith R.N., Yagita H., Akiba H., Yamazaki T., Azuma M., Iwai H., Khoury S.J. (2003). The programmed death-1 (PD-1) pathway regulates autoimmune diabetes in nonobese diabetic (NOD) mice. J. Exp. Med..

[68] Quandt Z., Young A., Anderson M. (2020). Immune checkpoint inhibitor diabetes mellitus: A novel form of autoimmune diabetes. Clin. Exp. Immunol..

[69] Mourad D., Azar N.S., Eid A.A., Azar S.T. (2021). Immune checkpoint inhibitor-induced diabetes mellitus: Potential role of t cells in the underlying mechanism. Int. J. Mol. Sci..

[70] Bluestone J.A., Anderson M., Herold K.C., Stamatouli A.M., Quandt Z., Perdigoto A.L., Clark P.L., Kluger H., Weiss S.A., Gettinger S. (2018). Collateral damage: Insulin-dependent diabetes induced with checkpoint inhibitors. Diabetes.

[71] De Filette J.M.K., Pen J.J., Decoster L., Vissers T., Bravenboer B., Van Der Auwera B.J., Gorus F.K., Roep B.O., Aspeslagh S., Neyns B. (2019). Immune checkpoint inhibitors and type 1 diabetes mellitus: A case report and systematic review. Eur. J. Endocrinol..

[72] Wu L., Tsang V., Menzies A.M., Sasson S.C., Carlino M.S., Brown D.A., Clifton-Bligh R., Gunton J.E. (2023). Risk Factors and Characteristics of Checkpoint Inhibitor–Associated Autoimmune Diabetes Mellitus (CIADM): A Systematic Review and Delineation From Type 1 Diabetes. Diabetes Care.

[73] Clotman K., Janssens K., Specenier P., Weets I., De Block C.E.M. (2018). Programmed Cell Death-1 Inhibitor-Induced Type 1 Diabetes Mellitus. J. Clin. Endocrinol. Metab..

[74] Zhang Z., Sharma R., Hamad L., Riebandt G., Attwood K. (2023). Incidence of diabetes mellitus in patients treated with immune checkpoint inhibitors (ICI) therapy—A comprehensive cancer center experience. Diabetes Res. Clin. Pract..

[75] Hatayama S., Kodama S., Kawana Y., Otake S., Sato D., Horiuchi T., Takahashi K., Kaneko K., Imai J., Katagiri H. (2022). Two cases with fulminant type 1 diabetes that developed long after cessation of immune checkpoint inhibitor treatment. J. Diabetes Investig..

[76] Cho Y.K., Jung C.H. (2023). Immune-Checkpoint Inhibitors-Induced Type 1 Diabetes Mellitus: From Its Molecular Mechanisms to Clinical Practice. Diabetes Metab. J..

[77] Imagawa A., Hanafusa T., Awata T., Ikegami H., Uchigata Y., Osawa H., Kawasaki E., Kawabata Y., Kobayashi T., Shimada A. (2012). Report of the Committee of the Japan Diabetes Society on the Research of Fulminant and Acute-onset Type 1 Diabetes Mellitus: New diagnostic criteria of fulminant type 1 diabetes mellitus (2012). J. Diabetes Investig..

[78] Zhou L., Yang S., Li Y., Xue C., Wan R. (2024). A comprehensive review of immune checkpoint inhibitor-related diabetes mellitus: Incidence, clinical features, management, and prognosis. Front. Immunol..

[79] Brahmer J.R., Abu-Sbeih H., Ascierto P.A., Brufsky J., Cappelli L.C., Cortazar F.B., Gerber D.E., Hamad L., Hansen E., Johnson D.B. (2021). Society for immunotherapy of cancer (sitc) clinical practice guideline on immune checkpoint inhibitor-related adverse events. J. Immunother. Cancer.

[80] Liao D., Liu C., Chen S., Liu F., Li W., Shangguan D., Yinguri S. (2023). Recent advances in immune checkpoint inhibitor-induced type 1 diabetes mellitus. Int. Immunopharmacol..

[81] Wu L., Tsang V., Clifton-Bligh R., Carlino M.S., Tse T., Huang Y., Oatley M., Cheung N.W., Long G.V., Menzies A.M. (2025). Hyperglycemia in patients treated with immune checkpoint inhibitors: Key clinical challenges and multidisciplinary consensus recommendations. J. Immunother. Cancer.

[82] Fujiwara Y., Takeda K., Yoshino T., Bando H., Arita K., Ishii N., Takahashi S. (2020). Infection risk with PI3K-AKT-mTOR pathway inhibitors and immune checkpoint inhibitors in patients with advanced solid tumours in phase I clinical trials. ESMO Open.

[83] Morelli T., Fujita K., Redelman-Sidi G., Elkington P.T. (2022). Infections due to dysregulated immunity: An emerging complication of cancer immunotherapy. Thorax.

[84] Papadakis M., Karniadakis I., Mazonakis N., Akinosoglou K., Tsioutis C., Spernovasilis N. (2023). Immune Checkpoint Inhibitors and Infection: What Is the Interplay?. Vivo.

[85] Gudiol C., Hicklen R.S., Okhuysen P.C., Malek A.E., Kontoyiannis D.P. (2022). Infections Simulating Immune Checkpoint Inhibitor Toxicities: Uncommon and Deceptive. Open Forum Infect. Dis..

[86] Grubbs J.A., Baddley J.W. (2014). Pneumocystis jirovecii pneumonia in patients receiving tumor-necrosis-factor-inhibitor therapy: Implications for chemoprophylaxis. Curr Rheumatol Rep..

[87] Petrelli F., Morelli A.M., Luciani A., Ghidini A., Solinas C. (2021). Risk of Infection with Immune Checkpoint Inhibitors: A Systematic Review and Meta-analysis. Target Oncol..

[88] Picchi H., Cavalcanti G.P., Orcurto A.S.S.F., Freire M.S., Tardomi N.S.C., Costa D.S.M.G.L.F., Barros S.V.M., Silva M.N.S., Reis V.M.A.P. (2018). Infectious complications associated with the use of immune checkpoint inhibitors in oncology: Reactivation of tuberculosis after anti PD-1 treatment. Clin. Microbiol. Infect..

[89] Redelman-Sidi G., Michielin O., Cervera C., Ribi C., Aguado J.M., Fernandez Ruiz M., Manuel O. (2018). ESCMID Study Group for Infections in Compromised Hosts (ESGICH) Consensus Document on the safety of targeted and biological therapies: An infectious diseases perspective (Immune checkpoint inhibitors, cell adhesion inhibitors, sphingosine-1-phosphate receptor modulators and proteasome inhibitors). Clin. Microbiol. Infect..

[90] Del Castillo M., Romero F.A., Argüello E., Kyi C., Postow M.A., Redelman-Sidi G. (2016). The Spectrum of Serious Infections Among Patients Receiving Immune Checkpoint Blockade for the Treatment of Melanoma. Clin. Infect. Dis..

[91] Lázár-Molnár E., Chen B., Sweeney K.A., Wang E.J., Liu W., Lin J., Porcelli S.A., Almo S.C., Nathenson S.G., Jacobs Jr W.R. (2010). Programmed death-1 (PD-1)–deficient mice are extraordinarily sensitive to tuberculosis. Proc. Natl. Acad. Sci. USA.

[92] Fujita K., Kim Y.H., Kanai O., Yoshida H., Mio T., Hirai T. (2019). Emerging concerns of infectious diseases in lung cancer patients receiving immune checkpoint inhibitor therapy. Respir. Med..

[93] Lo B., Fritz J.M., Su H.C., Uzel G., Jordan M.B., Lenardo M.J. (2016). CHAI and LATAIE: New genetic diseases of CTLA-4 checkpoint insufficiency. Blood.

[94] Gámez-Díaz L., August D., Stepensky P., Revel-Vil S., Seidel M.G., Noriko M., Moiro T., Worth A.J.J., Blessing J., Van de Veerdonk F. (2016). The extended phenotype of LPS-responsive beige-like anchor protein (LRBA) deficiency. J. Allergy Clin. Immunol..

[95] Abers M.S., Lionakis M.S. (2020). Infectious Complications of Immune Checkpoint Inhibitors. Infect. Dis. Clin. N. Am..

[96] Langan E.A., Graetz V., Allerheiligen J., Zillikens D., Rupp J., Terheyden P. (2020). Immune checkpoint inhibitors and tuberculosis: An old disease in a new context. Lancet Oncol..

[97] Zhang X., Zhou Y., Chen S., Fang W., Cai X., Zhang X., Zhao M., Zhang B., Jiang W., Lin Z. (2019). Hepatitis B virus reactivation in cancer patients with positive Hepatitis B surface antigen undergoing PD-1 inhibition. J. Immunother. Cancer.

[98] Babacan N.A., Tanvetyanon T. (2019). Superimposed Clostridium difficile Infection During Checkpoint Inhibitor Immunotherapy-induced Colitis. J. Immunother..

[99] Picasso R., Cozzi A., Picasso V., Zaottini F., Pistoia F., Perissi S., Martinoli C. (2023). Immune checkpoint inhibitor-related pneumonitis and COVID-19: A case-matched comparison of CT findings. Radiol. Med..

[100] Kyi C., Hellmann M.D., Wolchok J.D., Chapman P.B., Postow M.A. (2014). Opportunistic infections in patients treated with immunotherapy for cancer. J. Immunother. Cancer.

[101] Lasagna A., Arlunno B., Imarisio I. (2022). A case report of pulmonary nocardiosis during pembrolizumab: The emerging challenge of the infections on immunotherapy. Immunotherapy.

[102] Quartermain L., Buchan C.A., Kilabuk E., Wheatley-Price P. (2024). Pulmonary Nocardiosis in a Non-Small Cell Lung Cancer Patient Being Treated for Pembrolizumab-Associated Pneumonitis. Case Rep. Oncol..

[103] Jung J., Park S.Y., Park J.Y., Kim D., Lee K., Choi S. (2024). Reactivation of Varicella-Zoster Virus in Patients with Lung Cancer Receiving Immune Checkpoint Inhibitors: Retrospective Nationwide Population-Based Cohort Study from South Korea. Cancers.

[104] Jung J., Park S.Y., Jo H.B., Park J.Y., Kim D., Lee K., Choi S. (2025). Pneumocystis jirovecii pneumonia in patients with lung cancer receiving immune checkpoint inhibitors: A retrospective nationwide population-based cohort study from South Korea. Discov. Oncol..

[105] Ross J.A., Komoda K., Pal S., Dickter J., Salgia R., Dadwal S. (2022). Infectious complications of immune checkpoint inhibitors in solid organ malignancies. Cancer Med..

[106] Machado A.P., Correia L.B., da Silva J.B., de Melo A.C. (2023). The Safety of Immunosuppressants Used in the Treatment of Immune-Related Adverse Events due to Immune Checkpoint Inhibitors: A Systematic Review. J. Cancer.

[107] Franklin C., Rooms I., Fiedler M., Reis H., Milsch L., Herz S., Livingston E., Zimmer L., Schmid K.W., Dittmer U. (2017). Cytomegalovirus reactivation in patients with refractory checkpoint inhibitor-induced colitis. Eur. J. Cancer.

[108] Uchida N., Fujita K., Nakatani K., Mio T. (2017). Acute Progression of Aspergillosis in a Patient with Lung Cancer Receiving Nivolumab. Respirol. Case Rep..

[109] Del Rivero J., Cordes L.M., Klubo-Gwiezdzinska J., Madan R.A., Nieman L.K., Gulley J.L. (2020). Endocrine-Related Adverse Events Related to Immune Checkpoint Inhibitors: Proposed Algorithms for Management. Oncologist.

[110] Huang H.K., Wang J.H., Kao S.L. (2021). Risk of developing pneumonia associated with clinically diagnosed hypothyroidism: A nationwide population-based cohort study. Fam. Pract..

[111] Thorkildsen M.S., Mohus R.M., Åsvold B.O., Skei N.V., Nilsen T.I.L., Solligård E., Damas J.K., Gustad L.T. (2022). Thyroid function and risk of bloodstream infections: Results from the Norwegian prospective population-based HUNT Study. Clin. Endocrinol..

[112] Tan T.L., Rajeswaran H., Haddad S., Shahi A., Parvizi J. (2016). Increased Risk of Periprosthetic Joint Infections in Patients With Hypothyroidism Undergoing Total Joint Arthroplasty. J. Arthroplast..

[113] Ngaosuwan K., Johnston D.G., Godsland I.F., Cox J., Majeed A., Quint J.K., Oliver N., Robinson S. (2021). Increased Mortality Risk in Patients with Primary and Secondary Adrenal Insufficiency. J. Clin. Endocrinol. Metab..

[114] Ebrahimi F., Widmer A., Wagner U., Mueller B., Schuetz P., Christ-Crain M., Kutz A. (2019). Association of adrenal insufficiency with patient-oriented health-care outcomes in adult medical inpatients. Eur. J. Endocrinol..

[115] Bergthorsdottir R., Esposito D., Olsson D.S., Ragnarsson O., Dahlqvist P., Bensing S., Natman J., Johannsson G., Nyberg F. (2024). Increased risk of hospitalization, intensive care and death due to COVID-19 in patients with adrenal insufficiency: A Swedish nationwide study. J. Intern. Med..

[116] Janssen A.W.M., Stienstra R., Jaeger M., van Gool A.J., Joosten L.A.B., Netea M.G., Riksen N.P., Tack C.J. (2021). Understanding the increased risk of infections in diabetes: Innate and adaptive immune responses in type 1 diabetes. Metabolism.

[117] Chen X.H., Liu H.Q., Nie Q., Wang H., Xiang T. (2023). Causal relationship between type 1 diabetes mellitus and six high-frequency infectious diseases: A two-sample mendelian randomization study. Front. Endocrinol..

[118] Muller L.M.A.J., Gorter K.J., Hak E., Goudzwaard W.L., Schellevis F.G., Hoepelman A.I.M., Rutten G.E.H.M. (2005). Increased risk of common infections in patients with type 1 and type 2 diabetes mellitus. Clin. Infect. Dis..

[119] Critchley J.A., Carey I.M., Harris T., DeWilde S., Hosking F.J., Cook D.G. (2018). Glycemic control and risk of infections among people with type 1 or type 2 diabetes in a large primary care cohort study. Diabetes Care.

[120] Campochiaro C., Farina N., Tomelleri A., Ferrara R., Lazzari C., De Luca G., Bulotta A., Signorelli D., Palmisano A., Vignale D. (2021). Tocilizumab for the treatment of immune-related adverse events: A systematic literature review and a multicentre case series. Eur. J. Intern. Med..

[121] Guo S.B., Liu D.Y., Hu R., Zhou Z.Z., Meng Y., Li H.L., Huang W.J., Tian X.P. (2025). Immune-related adverse events of neoadjuvant immunotherapy in patients with perioperative cancer: A machine-learning-driven, decade-long informatics investigation. J. Immunother. Cancer.

